# Physics-Guided Residual Learning with Conditional Modulation for Quality Monitoring of Kiwifruit Juice During Pasteurization

**DOI:** 10.3390/foods15152627

**Published:** 2026-07-27

**Authors:** Yu Xia, Xinrui Hu, Yixuan Li, Wenbo Liu, Jie Kang, Wei Tang, Pengfei Jia, Shuai Zhang, Peng Wang, Min Xu

**Affiliations:** 1 School of Electrical and Control Engineering, Shaanxi University of Science and Technology, Longshuo Road, Weiyang District, Xi’an 710021, China; 2 School of Electrical Engineering, Guangxi University, Nanning 530004, China; 3 College of Food Engineering and Nutritional Science, Shaanxi Normal University, Chang’an West Road, Chang’an District, Xi’an 710119, China; 4 Xinjiang Changji Agricultural Science and Technology Park Agricultural Science and Technology Development Co., Ltd., Changji 831100, China; 5 College of Food Science, Southwest University, Chongqing 400715, China

**Keywords:** near-infrared spectroscopy, electronic nose, small-sample learning, temporal prediction, juice quality monitoring

## Abstract

Accurate prediction of quality indicators during juice pasteurization is challenged by the intricate coupling between temperature-dependent reaction kinetics and the limited availability of labeled data under laboratory-controlled pasteurization conditions. Here, we present a physics-guided residual learning framework that fuses near-infrared spectroscopy with electronic nose signals for dynamic quality monitoring. A first-order volatilization–saturation kinetic equation is embedded as a physical prior (PIRL), anchoring the prediction to known degradation behavior while reserving the residual learner for data-driven compensation of unmodeled deviations. To further incorporate process control information, a Feature-wise Linear Modulation-inspired conditional modulation (FiLM–ICM) mechanism is deployed, enabling temperature and time to act not as passive covariates but as active modulators that adaptively rescale spectral and olfactory feature responses. Validated on a kiwifruit juice pasteurization dataset under leave-one-trajectory-out cross-validation, the PIRL–FiLM framework consistently outperforms conventional chemometric baselines, achieving R^2^ improvements up to 0.030 and RPD gains exceeding 0.230. This hybrid strategy demonstrates that physical knowledge and data-driven learning are not competing but complementary, offering a robust and interpretable paradigm for process analytical technology under small-sample constraints. This framework offers a practical solution for quality monitoring in scenarios where large-scale labeled datasets are unavailable, such as in laboratory-scale process development and small-batch production.

## 1. Introduction

Visible–near-infrared (VIS–NIR) spectroscopy technology, with its significant advantages of non-destructiveness, rapid response, and simultaneous multi-component detection, has been established as the core analytical method for dynamic monitoring of agricultural product quality [1,2]. During temperature-driven food processing or storage, color indicators (a*, b*, C*, h°) and intrinsic quality indicators (such as pH value, sugar content Brix) can comprehensively reflect the evolution trajectory of the product’s overall quality [3,4]. If the quality changes at the next moment can be accurately predicted based on the spectral and sensor readings from previous moments, it will provide low-latency feedback signals for process control, enabling early intervention for abnormal conditions and effectively reducing losses [5,6].

In this study, transmission spectroscopy was employed for online monitoring of a laboratory-controlled pasteurization process. In the high-temperature and flowing simulated system, the spectral probe was configured to measure primarily the transmitted spectrum, rather than the specular reflection spectrum measured by a standard colorimeter under controlled conditions. L* (brightness), as a measure of specular reflection characteristics, is more dependent on the physical state of the surface (such as roughness and glossiness) than on the internal chemical composition [7]. Therefore, VIS–NIR, which is based on internal penetration characteristics, exhibits inherent physical limitations in capturing surface brightness information. Consequently, a*, b*, C*, and h° were selected as the color prediction targets, while L* was deliberately excluded [8]. This strategy aligns with the difference in spectral physical mechanisms for capturing surface versus internal information and also conforms to the actual constraints of industrial online monitoring [9].

However, in the laboratory stage of VIS–NIR chemometrics, the sample size is usually small, and under time-series conditions, both temperature and time must be considered simultaneously as external variables. If temperature and time are directly concatenated as additional features to the high-dimensional spectral vector, the temperature–time signals are often overwhelmed by the spectral dimensions, resulting in a decrease in prediction accuracy [10]. Moreover, the spectral feature dimension is as high as 2048, and direct modeling not only has high computational costs but also is prone to overfitting problems [11]. To solve the contradiction between high-dimensional data and small samples, this study builds a three-source fusion time-series prediction system around VIS–NIR and electronic nose (E-nose) [12].

To address the aforementioned limitations, the present study was designed with the following specific objectives [13]:

(1) To overcome the physical limitation of VIS–NIR in capturing surface brightness information, an E-nose was incorporated as a complementary sensing modality, and a multi-source data fusion strategy was established to integrate spectral and olfactory features for comprehensive color prediction [14,15].
(2) To resolve the high-dimensional small-sample dilemma and the issue of external variable suppression, a three-source fusion time-series prediction framework was developed, in which temperature and time variables were encoded as separate input streams rather than being concatenated directly with spectral features, thereby preventing their dilution by the high-dimensional spectral vector [16].
(3) To mitigate the overfitting risk and computational burden associated with 2048-dimensional spectral data, feature selection and dimensionality reduction techniques were applied prior to model construction, ensuring that the prediction models remained parsimonious and generalizable [1].

## 2. Materials and Methods

### 2.1. Materials

**Figure 1:**
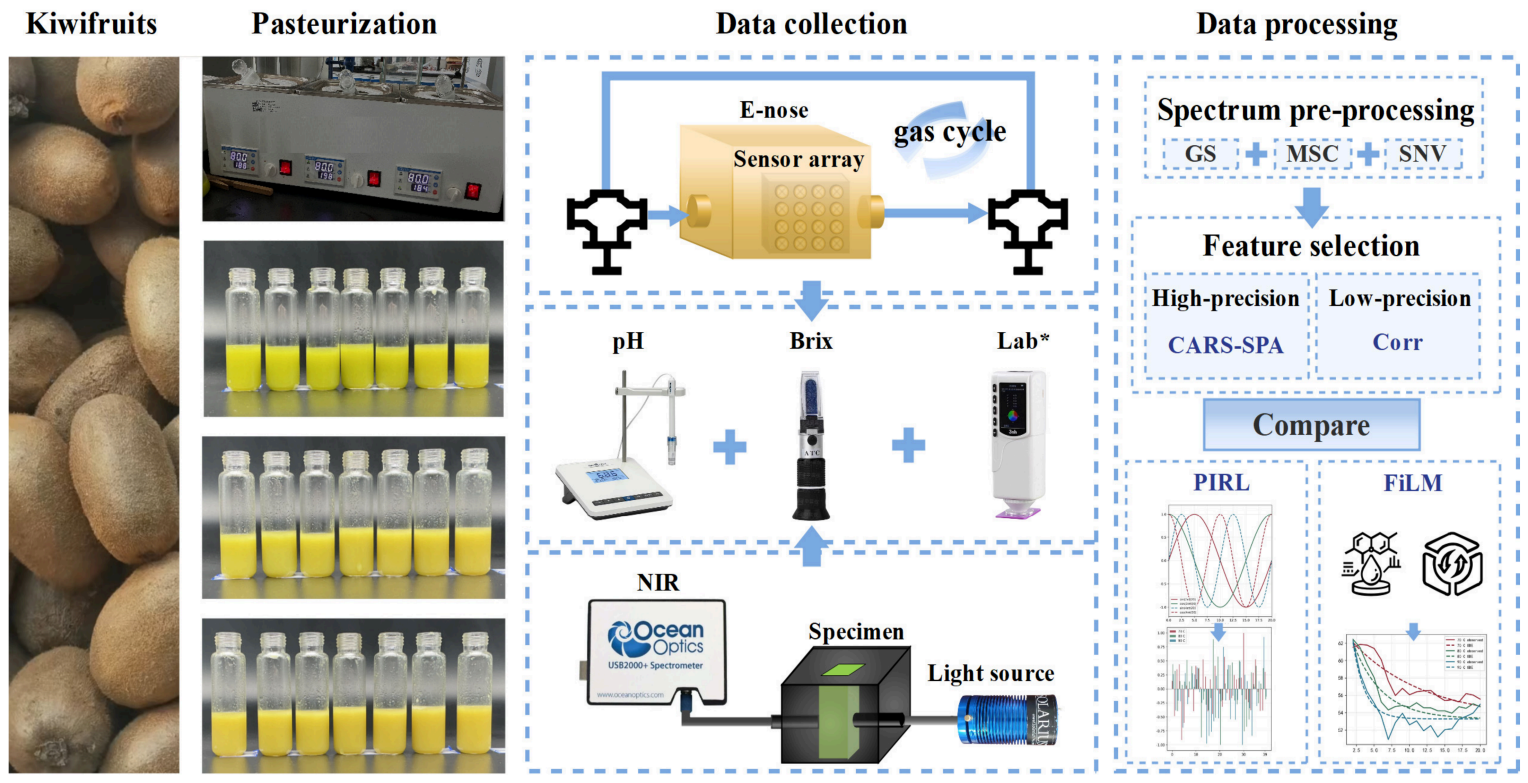
Materials and Methodology Flowchart.

The overall experimental workflow is illustrated in Figure 1. In August 2025, hand-picked ‘Cuixiang’ (CX) kiwifruits were collected from an orchard in Zhouzhi County, Shaanxi Province, China (108.22° E, 34.16° N). All selected fruits were at the optimal maturity stage, with soluble solids content of 15–17 °Brix, as determined using a BK8280 Brix meter (North-South Instrument Co., Ltd., Zhengzhou, Henan, China) and were free from visible damage. The pH values of the juice samples were measured using a PHS-3C pH meter (Shanghai INESA Scientific Instrument Co., Ltd., Shanghai, China). Color parameters of the juice samples were measured using an NR10QC color difference meter (Shenzhen 3nh Technology Co., Ltd., Shenzhen, Guangdong, China). The fruits were sliced and juiced, and 100 mL of juice was taken from each group for analysis. The juice samples were heated in constant-temperature water baths at 70, 80, and 90 °C to simulate pasteurization conditions. Samples were collected every minute from 0 to 20 min, resulting in 21 time points for each temperature condition.

For balanced model comparison, eight replicate trajectories were retained for each temperature, giving 24 trajectories in total (3 temperatures × 8 replicates). The eight replicate trajectories per temperature condition were prepared from the same batch of kiwifruit juice after homogenization and aliquoting. They should be considered technical replicates rather than independent biological replicates. Each trajectory contained 21 time points, corresponding to 504 original time-point records before temporal windowing. In the current temporal prediction setting, the measurements at the previous two time points were used to predict the quality values at the current time point. Therefore, the first two time points of each trajectory were used only as historical inputs, and the final supervised dataset contained 456 modeling samples (24 trajectories × 19 prediction time points). The 504 count includes the initial time points that serve only as historical inputs, while the 252 raw E-nose files were reduced to 12-dimensional features per time point and aligned with the 456 modeling samples by temperature–time combination. Thus, all models were built on the identical 456-sample dataset.

VIS–NIR transmittance spectra in the range of 339–1024 nm were collected using a USB2000+ spectrometer. The USB2000+ spectrometer (Ocean Optics, Largo, FL, USA) was configured with a spectral resolution of ~0.33 nm and an integration time of 500 ms. Each spectrum was averaged from 1 scan. Wavelength calibration was performed using a mercury-argon (HgAr) standard prior to each measurement session. All measurements were conducted at room temperature (25 ± 1 °C). Each VIS–NIR spectrum contained 2048 wavelength variables.

The E-nose system was a self-developed 16-sensor array, with each acquisition cycle lasting 2 min, including 1 min of sweep and 1 min of measurement. A total of 252 valid E-nose files were used, corresponding to 8 E-nose groups, 21 time points, and 3 temperature conditions. It should be noted that the E-nose was used as a supplementary information source for data fusion with VIS–NIR spectra, rather than as the primary input for standalone prediction. Detailed hardware specifications (sensor types, sensitivity, selectivity) were therefore not the focus of this study. The E-nose responses were baseline-corrected by subtracting the initial reading before each measurement cycle to minimize drift.

All data processing, model training, and evaluation were implemented in Python 3.9.13 (Python Software Foundation, Wilmington, DE, USA) using the Visual Studio Code (version 1.129) environment (Microsoft Corp., Redmond, WA, USA). The following Python libraries were used: NumPy (NumPy developers, Austin, TX, USA), pandas (pandas developers, Austin, TX, USA), and scikit-learn (scikit-learn developers, Paris, France).

For each temperature–time combination, E-nose descriptors were averaged and reduced to 6 dimensions by PCA. Due to hardware limitations, each sample could only be measured by a single E-nose device sequentially at each time point, making continuous sampling at the exact same moment unfeasible. Additionally, even sensors of the same type may exhibit batch-to-batch variability. To mitigate these issues, the E-nose responses were averaged across the eight replicates for each temperature–time combination to obtain representative olfactory features. This pragmatic approach was adopted to maximize the utility of the available hardware; it should not be considered a standardized measurement protocol.

In the fused dataset, each modeling sample contained two VIS–NIR spectra from the previous two time points and two corresponding 6-dimensional E-nose feature vectors. Thus, the current fused dataset contained 456 samples with 4111 input variables, including 4096 VIS–NIR variables, 12 E-nose variables, and 3 temperature–time covariates.

### 2.2. Dataset Construction

The research objective is to predict the quality indicators for the next minute based on the spectral data from the previous two minutes and the E-nose signals. A time series sliding window is constructed with (context, horizon) = (2, 1): For each trajectory (same temperature and sample number), traverse in chronological order, and use the VIS–NIR features at t − 2 and t − 1 to predict the values of a*, b*, C*, h°, pH, and Brix at t. Sliding windows with missing or discontinuous data are discarded.

Two control experiments were designed in this study, as summarized in Table 1.

Both groups of experiments use the same data partitioning and evaluation criteria to ensure the fairness of the comparison, serving as the unified evaluation basis for subsequent baseline models and innovative methods.

With 24 independent trajectories and 456 modeling samples, the dataset is relatively small but representative of typical chemometric studies in food processing. To mitigate overfitting, feature selection reduced the original > 4000 variables to 83–95 input dimensions, LOSO cross-validation was applied to prevent data leakage, and the PIRL prior serves as an implicit regularizer. The use of only 24 trajectories (3 temperatures × 8 replicates) limits the assessment of between-batch and between-cultivar variability, and we acknowledge this limitation in Section 3.6.

### 2.3. Model and Algorithm

This study first compared seven wavelength selection methods, including correlation analysis (Corr), variable importance in projection (VIP), uninformative variable elimination (UVE), competitive adaptive reweighted sampling (CARS), successive projections algorithm (SPA), the hybrid CARS–SPA strategy, and interval partial least squares (iPLS), combined with five commonly used regression models, namely partial least squares regression (PLS), support vector regression (SVR), random forest (RF), extreme gradient boosting (XGB), and Gaussian process regression (GPR). These models cover representative linear, kernel-based, tree-ensemble, and Bayesian non-parametric regression families. Based on the overall prediction performance, the optimal combination was selected for subsequent modeling and comparison.

Based on this, two innovative methods were introduced: one is Physical Prior Residual Learning (PIRL) [17], which uses the volatility-saturation kinetic equation as the prior and explicitly injects the physical laws of temperature and time into the prediction process; the other is Temperature–Time Feature-wise Linear Modulation (FiLM) modulation, which uses conditional adaptive feature transformation to dynamically control the response intensity of spectral features to temperature and time with modulation coefficients [18]. To verify the effectiveness of the two innovative methods at different baseline levels, two sets of comparative experiments were designed: one is the high-accuracy baseline group based on CARS–SPA + GPR, and the other is the low-accuracy baseline group based on Corr + GPR. They, respectively, studied the optimization effects of the two optimization algorithms (PIRL, FiLM) and data fusion (VIS–NIR + E-nose) under the two conditions. Through a systematic comparison of five ablation control experiments (C0: no time–temperature covariates, C1: add time–temperature covariates, C2: only PIRL, C3: only FiLM, C4: PIRL + FiLM fusion), the independent contributions and synergistic effects of physical prior injection and conditional modulation, as well as the gain differences of data fusion at different baseline levels, were revealed.

Finally, through R^2^ analysis of the five ablation experiments (C0 to C4) on the prediction accuracy changes in the VIS–NIR-only and VIS–NIR + E-nose data fusion paths, and RPD analysis of the generalization ability differences in the same groups and paths, the comprehensive effect of the optimization algorithm and data fusion was evaluated [19]. The R^2^ and RPD results were obtained under strict LOSO cross-validation, which provides a more realistic estimate of model generalizability than traditional train-test splits.

PIRL embeds domain knowledge in the prediction model in the form of physical equations. In the simulation of pasteurization process, the changes in color parameters and quality indicators do not occur randomly but follow the basic chemical kinetic laws, namely the first-order chemical kinetic model [20]. Based on this assumption, PIRL first calculates the physical prior trend term according to the temperature-dependent kinetic equation, and then only allows the model to learn the residual part that the prior cannot capture. This strategy of adding first and then learning ensures the physical consistency of the prediction results and gives the model the ability to capture personalized deviations from data, effectively alleviating the overfitting problem in the case of small sample conditions [3].

The first-order chemical kinetic equation was used as the physical prior:

$$\frac{dG}{dt}=k(T)\cdot (G_{eq}-G)$$

where k(T) represents the temperature-dependent reaction rate constant, $G_{eq}$ denotes the equilibrium final value, and G is the target parameter value.

$$k(T)=k_{ref}\cdot e^{-\frac{E_{a}}{R}\left(\frac{1}{T}-\frac{1}{T_{ref}}\right)}$$

where $k_{ref}$ is the rate constant at the reference temperature, $E_{a}$ represents the activation energy (a characteristic parameter for a specific reaction), T represents the absolute temperature (70 °C, 80 °C, 90 °C), and $T_{ref}$ refers to the reference temperature, which is 70 °C = 343.15 K. R is fixed at 8.314, a universal constant [21]. Solving this differential equation yields the prior prediction at time *t*:

$${\hat{G}}_{prior}(t)=G_{eq}-(G_{eq}-G_{0})\cdot e^{-k(T)\cdot t}$$

where $G_{0}$ represents the initial value (the response value at *t* = 0), and ${\hat{G}}_{prior}(t)$ represents the physical model prediction value at time *t*.

α is the weight that allows the model to learn the physical prior, preventing negative impacts when the physical model is not accurately fitted. An adaptive parameter α is used. If the quality of the physical prior is high, α is close to 1; if the quality of the physical prior is low, α will decrease. α is the optimal weight in the least squares sense, and makes *α*⋅$G_{prior}$ become the orthogonal projection of *y* on $G_{prior}$.

$$\alpha =\frac{\langle G_{prior},y\rangle }{\langle G_{prior},G_{prior}\rangle }$$

where *y* represents the true value, and $G_{prior}$ represents the physical prior value.

The final prediction is the sum of the physical prior and the GPR residual prediction:

$$\hat{y}=y_{res}+\alpha \cdot G_{prior}$$

where $y_{res}$ represents the residual function predicted by GPR:

$$y_{res}\;=\;y\;-\;\alpha \cdot G_{prior}$$

FiLM is a conditional-dependent feature modulation mechanism. Its core idea lies in performing element-wise affine transformations on features through conditional vectors. This mechanism was proposed by Perez et al. in 2018 and initially applied in the field of conditional image generation [22]. It achieves dynamic adjustment of features at the feature level by learning modulation parameters. The mathematical expression of the standard FiLM includes multiplicative coefficients β(c) and additive bias γ(c), both of which are learned from the conditional vector c through neural networks, ultimately achieving the feature transformation of $\hat{x}=\beta (c)⊙x+\gamma (c)$. This mechanism enables the model to flexibly adjust feature representations according to different conditions and has demonstrated excellent performance in tasks such as few-shot learning and image generation.

In this study, the conditional modulation idea of FiLM was drawn upon and innovatively adapted to the traditional machine learning framework. In the construction of the conditional vector, a strategy combining temperature linear normalization (70/80/90 °C encoded as [−1, 0, 1]) and time sine–cosine encoding (fundamental frequency and double frequency) was adopted to construct a 5-dimensional conditional signal capable of describing the heating process. In the feature modulation stage, an outer product operation was performed on the first 10 core features and the conditional vector to generate 50-dimensional modulation features, thereby achieving temperature–time-dependent modulation at the feature level. Finally, by concatenating the original features (40 dimensions), the conditional vector (5 dimensions), and the modulation features (50 dimensions), a 95-dimensional enhanced feature space was formed. This design required no training of additional neural network parameters and achieved conditional-dependent feature transformation through pure feature engineering, while maintaining model simplicity and effectively improving cross-temperature prediction performance. Accordingly, we refer to this as “FiLM-inspired conditional modulation (FiLM-ICM)” throughout the manuscript.

Compared with the standard FiLM-ICM, the implementation of this study has significant characteristics: no need to learn modulation parameters, avoiding the complexity brought by neural network training; compatibility with traditional machine learning frameworks, suitable for small-sample chemical metrology scenarios; achieving dynamic adjustment of features at the temperature–time level through outer product modulation.

The fusion of PIRL and FiLM-ICM is a dual-level complementary strategy. FiLM-ICM realizes temperature–time dynamic modulation at the feature level, while PIRL uses the physical prior fitted by the first-order kinetic equation as the basis for prediction, and the GPR model focuses on learning the residuals between the true values and the physical predictions. The final prediction result is a linear combination of the physical prior and the residuals. This fusion enables FiLM-ICM to capture temperature–time change patterns at the feature level and PIRL to correct system deviations at the prediction level, with the two working collaboratively.

## 3. Results and Discussion

### 3.1. Spectral Preprocessing

**Figure 2:**
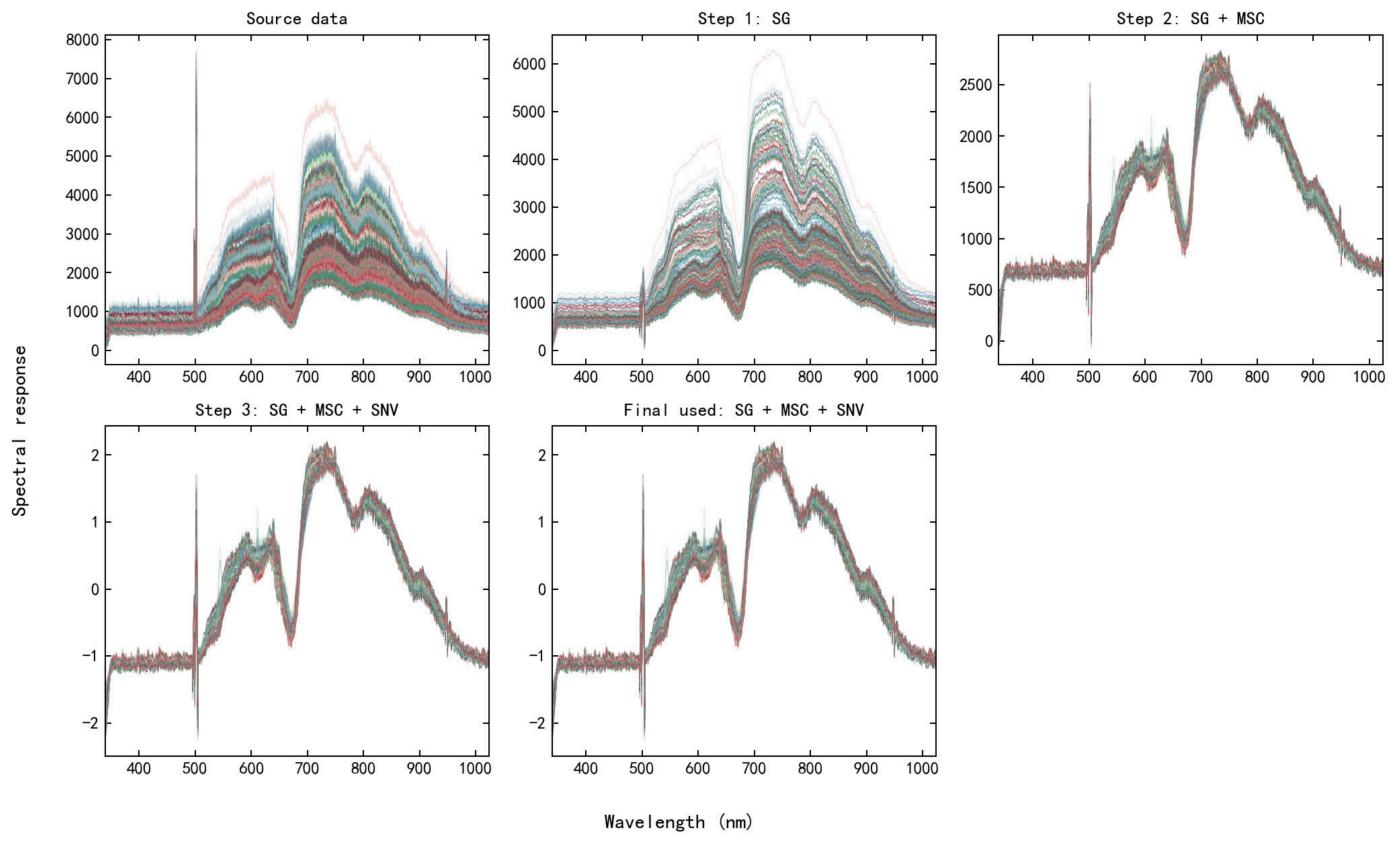
Spectral Preprocessing Result Plot.

The original VIS–NIR transmission spectra were only subjected to dark current correction (black calibration): Before each measurement, the light source was turned off to collect the dark current signal, which was used to subtract the dark noise of the detector and environmental light interference. The spectral data were sequentially smoothed using Savitzky-Golay (window 15, order 3), multi-scattering correction (MSC, with the mean spectrum as the reference), and standard normal variable transformation (SNV). The combination of these three steps can eliminate baseline drift, scattering effect, and amplitude differences between samples, obtaining a stable spectral characteristic basis. The wavelength comparison diagrams of the three stages are shown in Figure 2 below.

The combination of SG smoothing, MSC, and SNV was selected after comparing multiple preprocessing strategies, including single methods and various combinations (e.g., SG alone, SG + SNV, MSC + SNV, SG + SNV + MSC). This combination yielded the lowest prediction error on the validation set and was therefore adopted as the optimal preprocessing scheme.

**Figure 3:**
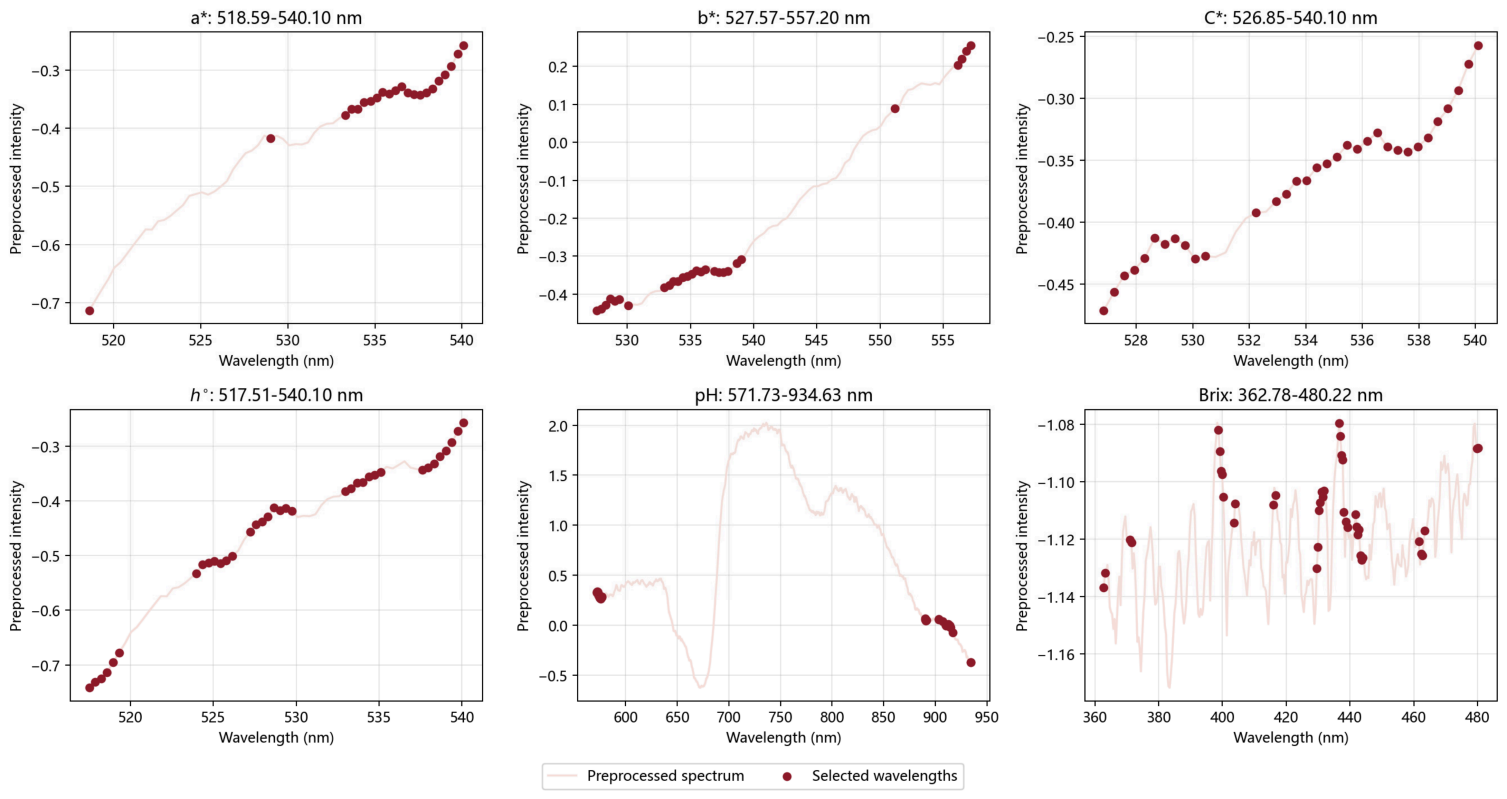
Feature Spectrum Plot.

From the perspective of the characteristic wavelength distribution in Figure 3, the characteristic wavelengths of color parameters (a*, b*, C*, h°) are completely concentrated in the visible light range of 585–641 nm, while pH and Brix shows a dual-region distribution characteristic in both visible light and VIS–NIR regions. This difference stems from the fundamentally different spectral response mechanisms of the two types of parameters.

Regarding the color parameters, the selected wavelengths were mainly distributed within the visible-light region, extending approximately from 435 to 780 nm. This distribution is consistent with the optical basis of the International Commission on Illumination (CIE) Lab* color system, which describes color perception based on human visual responses in the visible range [23]. In this study, the selected wavelengths for a*, b*, C*, and h° were 436.23–777.70 nm, 435.49–778.68 nm, 435.86–774.77 nm, and 436.60–779.98 nm, respectively. These wavelength regions cover the major absorption bands associated with fruit pigments, including carotenoids in the blue-green region, anthocyanin-related absorption around the green region, and chlorophyll-related absorption in the red region [24,25]. Therefore, the wavelength distribution of the color parameters indicates that their prediction mainly depended on visible absorption caused by pigment composition and color transformation in kiwifruit juice. This result supports the interpretation that color attributes are direct optical responses of the visible spectral region rather than being primarily governed by VIS–NIR molecular vibration information.

Compared with the color parameters, pH and Brix exhibited broader spectral distributions, suggesting more complex physicochemical response mechanisms. The selected wavelengths for pH ranged from 354.11 to 997.09 nm, covering both the visible and VIS–NIR regions. This indicates that pH prediction may involve not only pigment-related visible absorption changes caused by acid–pigment interactions, but also VIS–NIR responses associated with O–H and C=O groups in organic acids and water [4]. Similarly, the selected wavelengths for Brix ranged from 437.71 to 948.38 nm, showing that soluble solids prediction was not limited to a single visible or VIS–NIR band. The visible wavelengths may reflect changes in scattering, turbidity, and pigment background caused by soluble components, whereas the longer wavelengths may contain weak VIS–NIR information related to sugar-associated C–H and O–H bonds [26]. Therefore, unlike the color parameters, pH and Brix require broader spectral information, reflecting their combined dependence on optical scattering, pigment background, and molecular absorption characteristics.

### 3.2. Feature Extraction of E-Nose

After preprocessing the 16-channel sensor time series at each time point, principal component analysis (PCA) was employed for dimensionality reduction. Specifically, the mean response, standard deviation of response, and slope of response of the 16 sensors are regarded as three types of statistical features, each of which is considered a 16-dimensional vector and undergoes independent PCA transformation, ultimately being reduced to 2 dimensions. Thus, each time point has 6-dimensional E-nose features (mean PCA 2D + standard deviation PCA 2D + slope PCA 2D). This method effectively reduces the dimensionality of the E-nose features while retaining the core information such as sensor response intensity, fluctuation amplitude, and drift trend [27]. It also alleviates the overfitting risk under the condition of small sample size and lays the foundation for subsequent feature-level fusion with VIS–NIR.

### 3.3. Optimal Model Selection

The optimal modeling scheme suitable for spectral data was selected through this study. A systematic comparison was made among 7 feature selection methods and 5 regression algorithms. The feature selection methods included Corr, VIP, UVE, CARS, SPA, combined screening of CARS–SPA, and iPLS; the regression algorithms include GPR, PLS, RF, SVR, and XGB.

**Figure 4:**
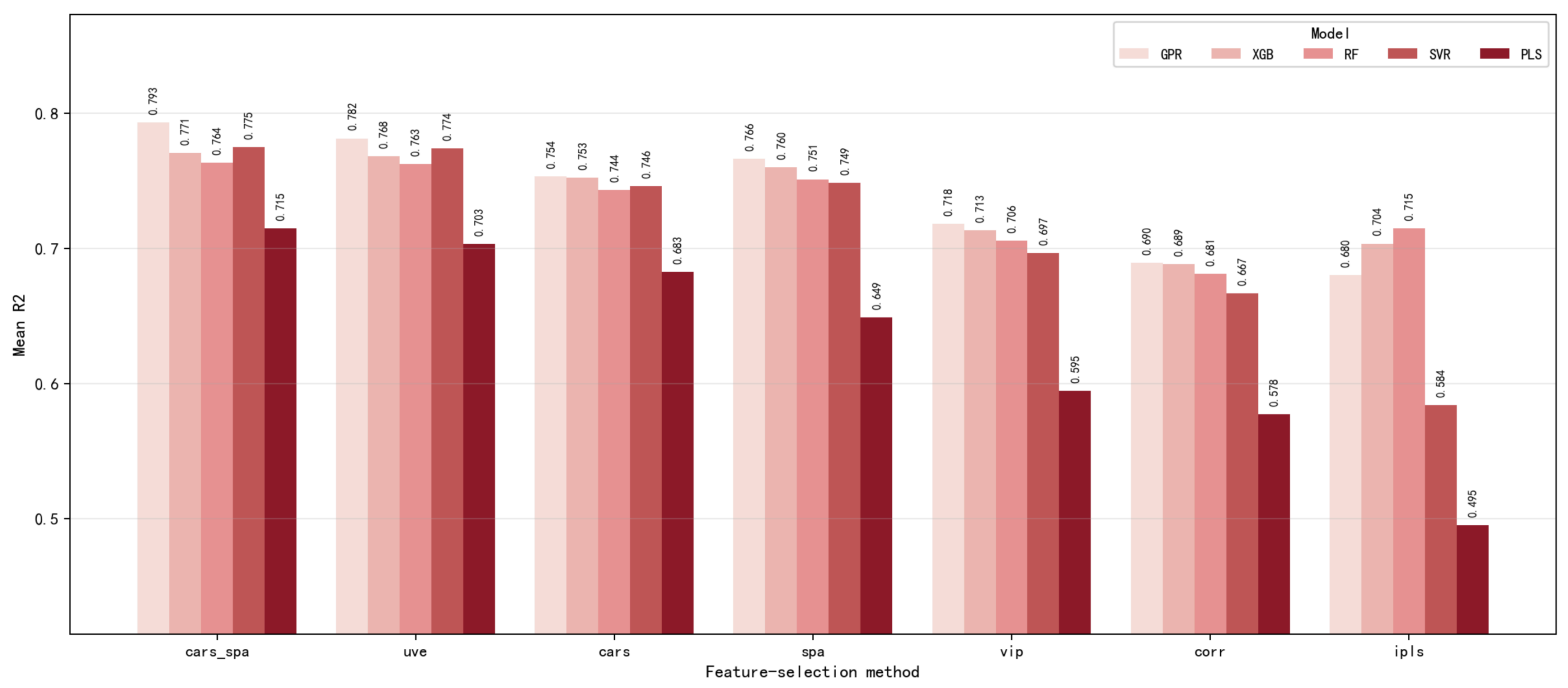
Mean R^2^ by feature selection method and algorithm.

The mean R^2^ across the six prediction targets was used as the comprehensive evaluation index. The results showed that both the feature selection strategy and the regression algorithm markedly affected prediction performance. Among all combinations, CARS–SPA combined with GPR achieved the highest mean R^2^ of 0.7934 and a mean RPD of 2.29, followed by UVE-GPR with a mean R^2^ of 0.7816. At the feature selection level, CARS–SPA and UVE performed better overall than CARS, SPA, VIP, Corr, and iPLS, indicating that hybrid or stability-based wavelength selection was more suitable for this dataset. At the model level, GPR showed the best overall performance, followed by XGB and RF, whereas PLS produced the weakest results, likely due to its linear modeling assumption, as illustrated in Figure 4. Among the six quality attributes, a* was the easiest to predict, followed by h°, pH, b*, and C*, while Brix showed the lowest mean R^2^, suggesting a weaker or more indirect spectral response. Therefore, the CARS–SPA wavelength selection strategy combined with GPR was selected as the optimal spectral modeling scheme for subsequent analysis, as summarized in Table 2.

**Figure 5:**
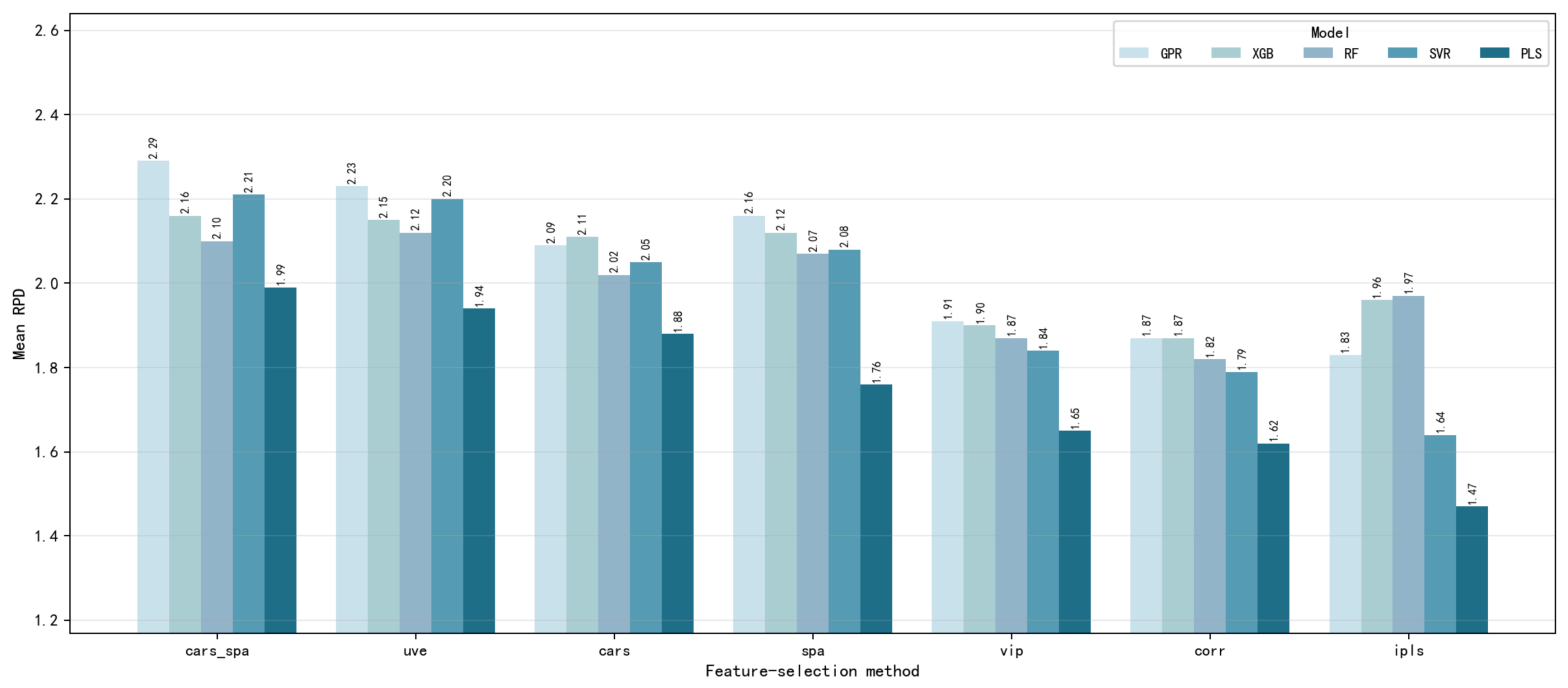
Mean RPD by feature selection method and algorithm.

Under the GPR model, the CARS–SPA method achieved the highest RPD value (2.29), exceeding the commonly accepted threshold for excellent prediction performance in chemometrics (RPD > 2.0), as shown in Figure 5 and Table 3. It was followed by UVE (2.23), SPA (2.16), and CARS (2.09), all of which also reached the excellent level. In contrast, VIP, Corr, and iPLS produced lower RPD values of 1.91, 1.87, and 1.83, respectively.

### 3.4. Model Optimization

**Figure 6:**
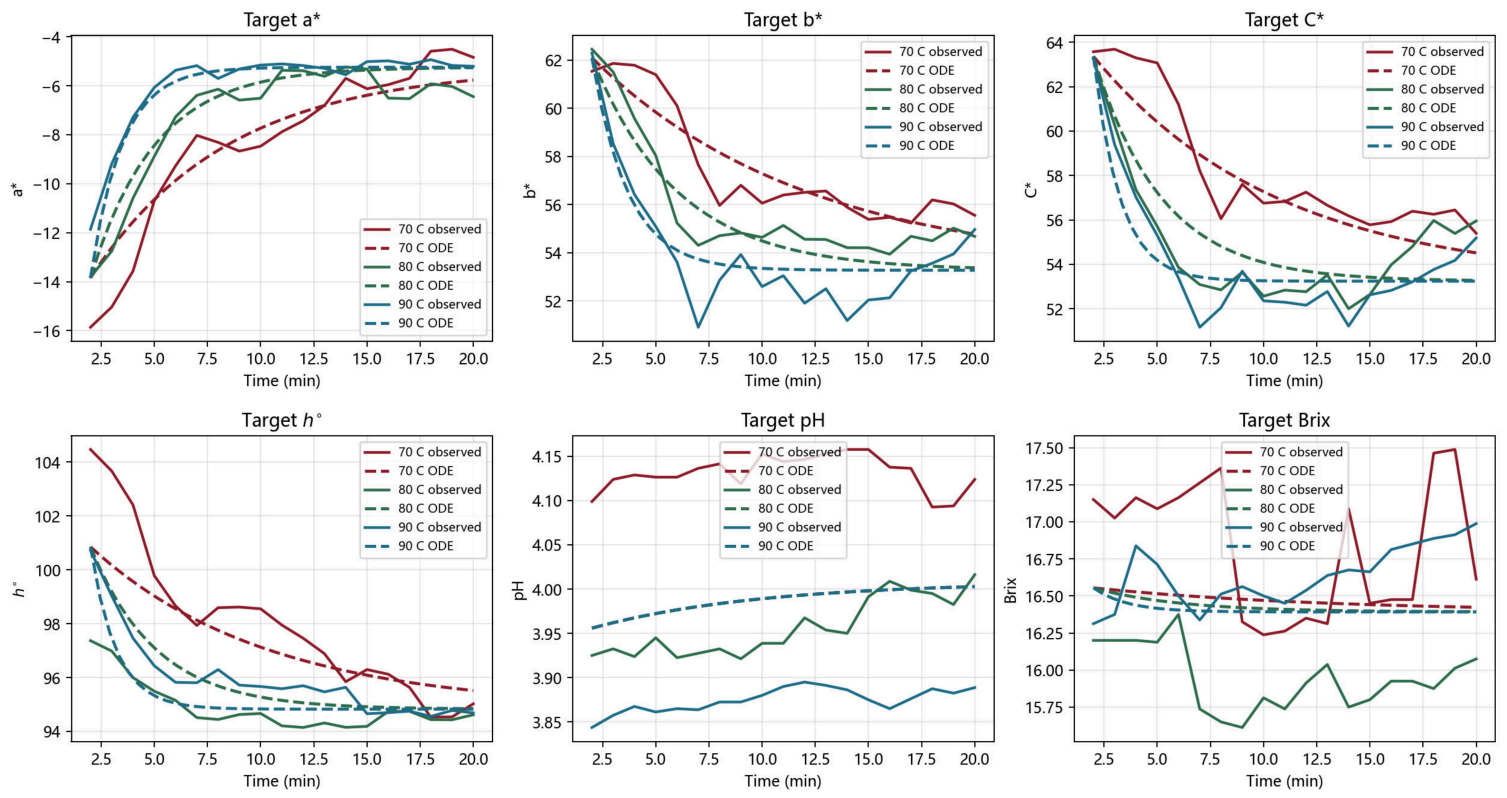
PIRL prior curves and observed means.

The first-order chemical kinetics equation was employed to establish a physical prior framework. Nonlinear fitting of the experimental data was performed using the differential evolution algorithm, and the kinetic parameters (reaction rate constant *k_0_*, activation energy *Ea*, and equilibrium value *G_eq_*) were estimated for each quality indicator, thereby generating physical priors under various temperature–time conditions, as illustrated in Figure 6. On this basis, a PIRL framework was constructed, in which the fitted physical laws were used as the trend term, and the machine learning model was designed to learn only the residuals between the experimental data and the prior trends. This approach enabled the capture of sample-specific deviations while retaining the physical constraints as a regularization backbone.

The first-order kinetic model was fitted to the experimental data for each quality indicator. The fitting R^2^ values were 0.913, 0.881, 0.841, 0.705, 0.014, and 0.039 for a*, b*, C*, h°, pH, and Brix, respectively, with corresponding RMSE values of 0.806, 1.001, 1.355, 1.260, 0.109, and 0.475.

Pure physical priors alone cannot independently achieve the predictive accuracy of chemical kinetics, but as the trend term in residual learning, they provide global guidance on temperature and time for subsequent machine learning, alleviating the difficulty of learning time–temperature dependence from scratch in small samples.

**Figure 7:**
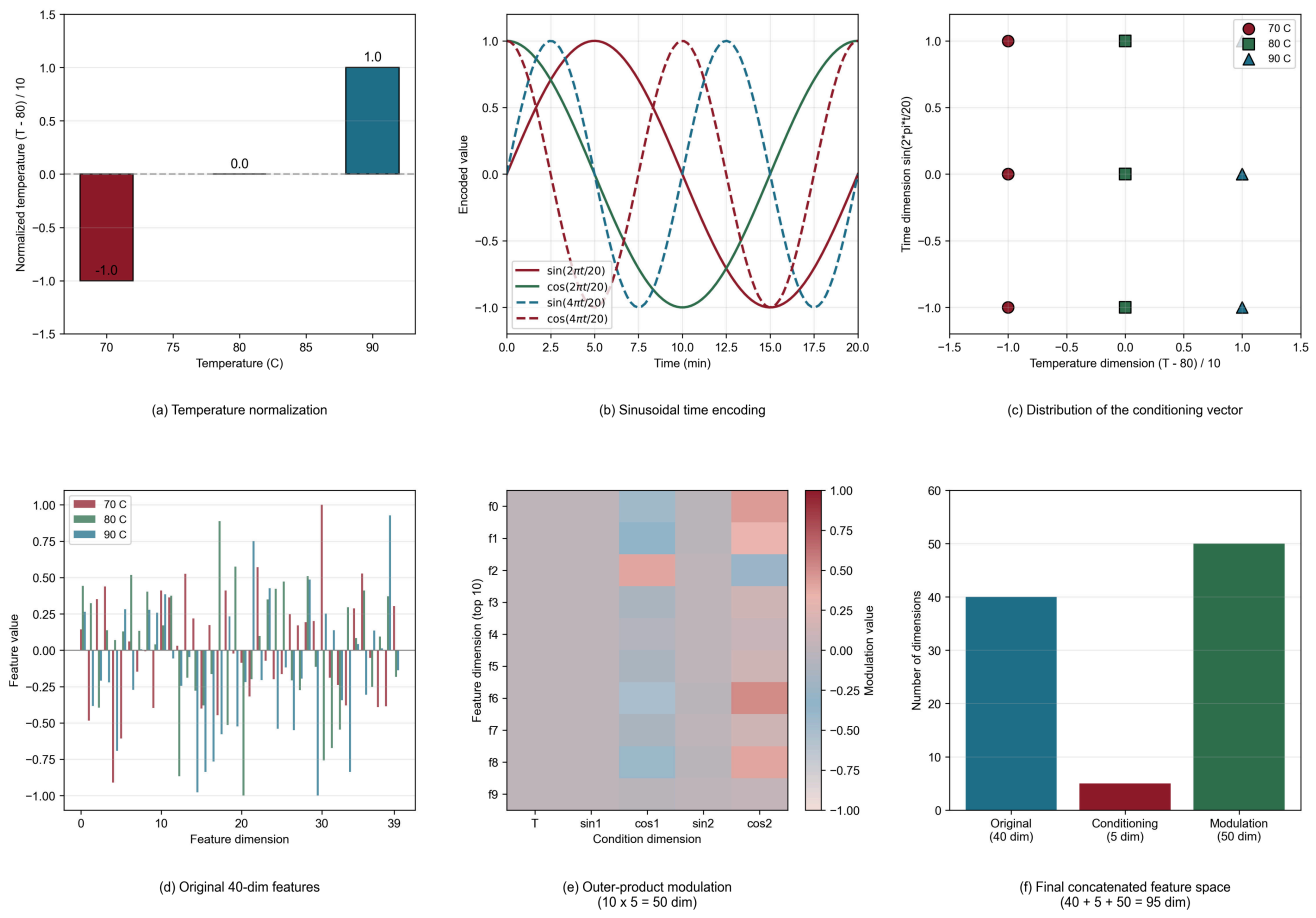
FiLM-ICM Linear Modulation Strategy Diagram.

This method is also applicable to low-dimensional sensor scenarios: even if the original feature dimension is limited, by constructing conditional vectors and modulating feature outer products, the feature space can still be effectively expanded and time–temperature dynamic information can be injected. For any low-dimensional sensor data, the core features can be extracted first through statistical descriptions (such as mean, standard deviation, slope, etc.) or dimensionality reduction methods, and then the FiLM-ICM mechanism is applied for feature increment—concatenating the normalized temperature values with the sine and cosine encoding of time into the conditional vector, selecting key features for outer product modulation, and finally forming an enhanced feature set that includes the original features, conditional vectors, and modulated features, as illustrated in Figure 7. This strategy provides an effective feature enhancement approach for low-dimensional sensor data, without relying on large-scale feature engineering to improve the model’s adaptability to dynamic environmental changes.

### 3.5. Prediction Results

**Figure 8:**
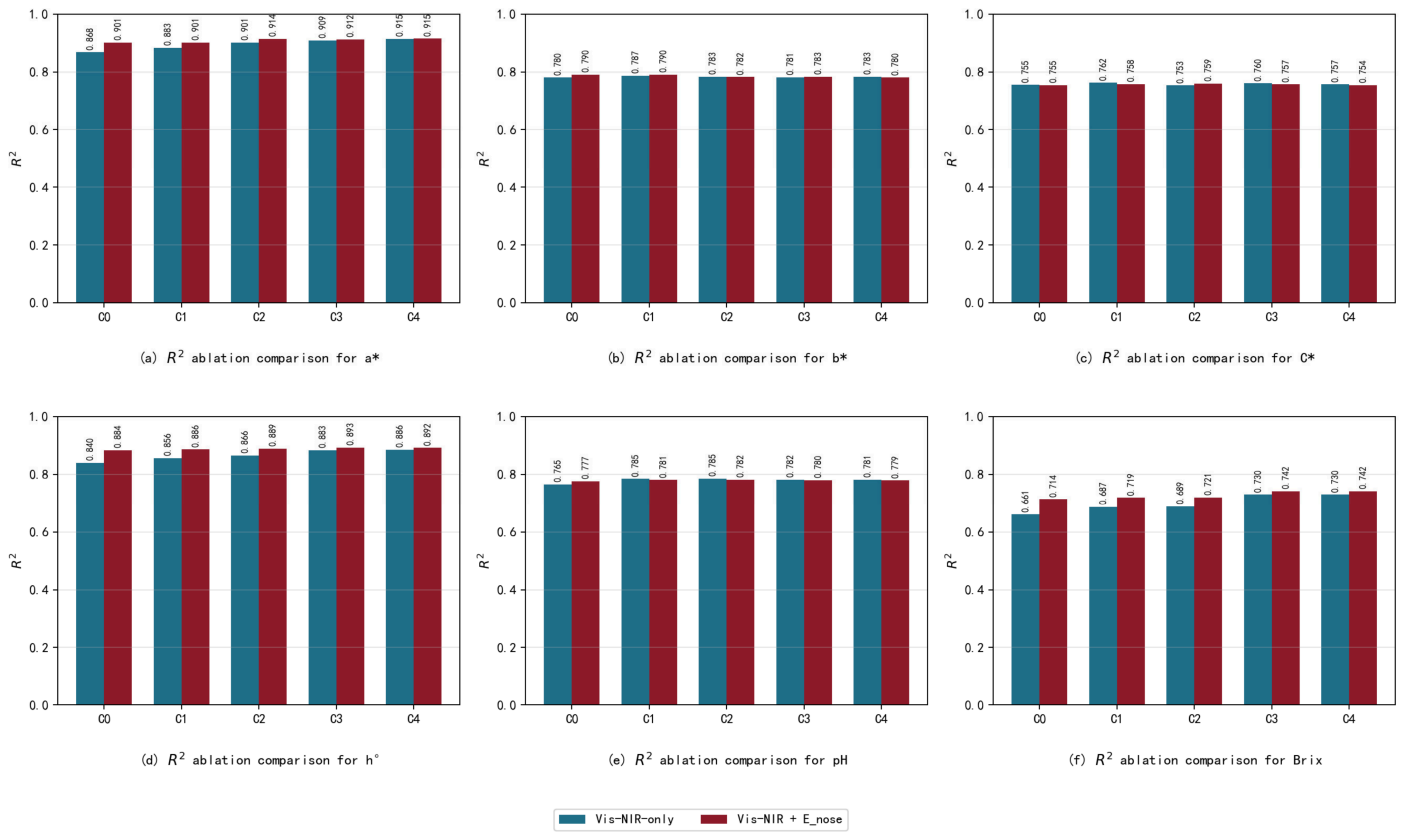
Accuracy comparison chart of the ablation group.

Leave-one-out cross-validation by trajectory grouping (LOSO) was adopted as the main validation strategy: each time, an independent trajectory was left out as the test set [28]. To further analyze the contribution of different optimization modules to model performance, the results of the ablation experiments were compared from two aspects. Firstly, by comparing the average R^2^ of each ablation group under the two modeling paths (VIS–NIR-only and fusion), the impacts of E-nose data fusion, the introduction of temperature and time information, PIRL, and FiLM-ICM conditional modulation on prediction accuracy were evaluated. Secondly, by combining the average RPD index, the generalization ability and stability of the model on unknown samples were examined. By observing the changes in R^2^ and RPD simultaneously, the mechanism of each module was more comprehensively judged: R^2^ mainly reflected the fitting accuracy of the model to the current test data, while RPD emphasized the magnitude of the model’s prediction error relative to the sample variance, thus better reflecting the practical application reliability of the model. Based on this idea, the performance of the five ablation groups from C0 to C4 under the two input paths was discussed separately in Figure 8.

According to the ablation results, C0 achieved a six-parameter mean R^2^ of 0.778 using VIS–NIR data alone. Incorporating E-nose information increased the mean R^2^ to 0.803, corresponding to an improvement of 2.5 percentage points and demonstrating the benefit of multisource data fusion. After directly introducing temperature and time variables (C1), the mean R^2^ increased to 0.793 for the VIS–NIR path and 0.806 for the fusion path. Compared with C0, these increases were 1.5 and 0.3 percentage points, respectively, indicating that direct concatenation provided only limited additional improvement, particularly after data fusion.

**Figure 9:**
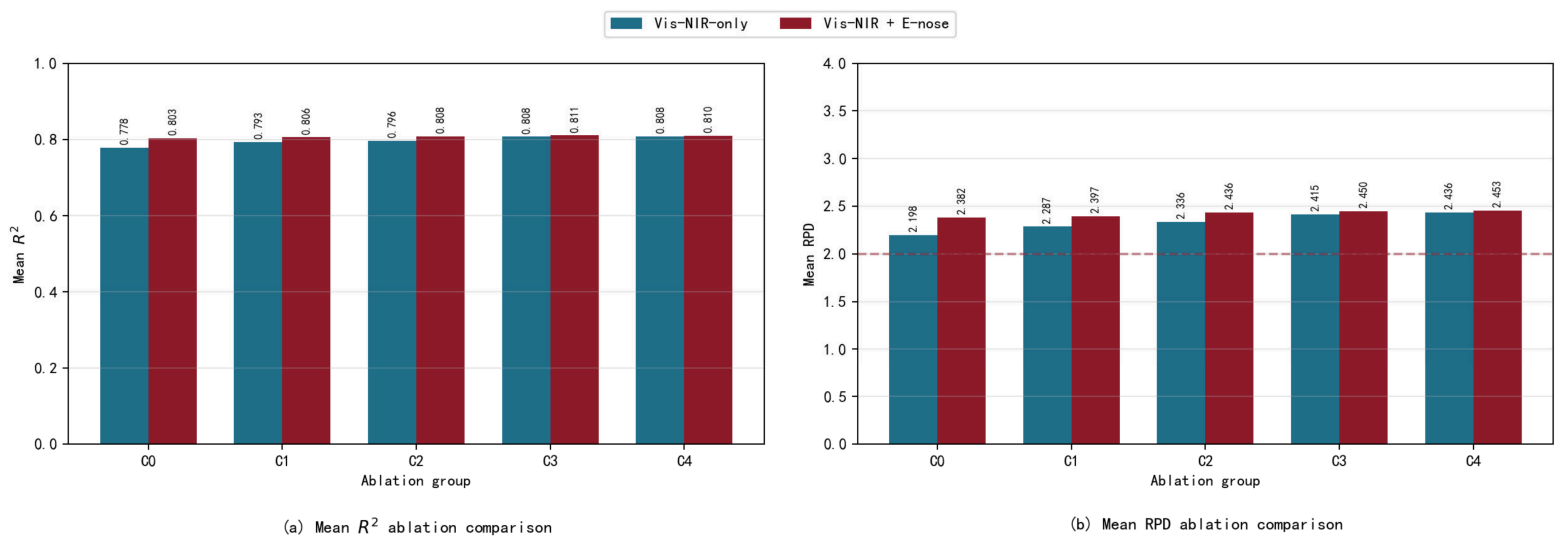
VIS–NIR data and the R^2^ of Fusion compared with RPD.

With PIRL (C2), the mean R^2^ reached 0.796 for VIS–NIR and 0.808 for fusion. FiLM-ICM modulation (C3) further improved the results to 0.808 and 0.811, respectively; compared with C1, the VIS–NIR result increased by 1.4 percentage points, demonstrating the effectiveness of conditional modulation. The combined PIRL–FiLM-ICM configuration (C4) produced mean R^2^ values of 0.808 for VIS–NIR and 0.810 for fusion. Although C4 did not outperform C3, its comparable performance indicates that the two mechanisms can be integrated without substantial performance degradation, as shown in Figure 9.

Comparison of the mean R^2^ and RPD values showed that the optimization strategies produced a modest improvement in prediction accuracy but a more pronounced enhancement in model generalization. For the VIS–NIR-only path, the mean R^2^ increased from 0.778 for C0 to 0.808 for C4, representing an improvement of 0.030. Meanwhile, the mean RPD increased from 2.198 to 2.436, an improvement of 0.238. These results indicate that the optimization modules primarily improved the robustness and generalization performance of the VIS–NIR-based model.

A similar but weaker trend was observed for the fusion path. From C0 to C4, the mean R^2^ increased from 0.803 to 0.810, while the mean RPD increased from 2.382 to 2.453. The highest fusion R^2^ was obtained by C3 (0.811), whereas C4 achieved the highest RPD (2.453). Thus, combining PIRL with temperature–time FiLM-ICM modulation did not substantially improve fitting accuracy over FiLM-ICM alone, but it slightly enhanced generalization performance. Overall, these modules appear to help the model learn more stable and transferable feature representations rather than merely improving goodness of fit.

**Figure 10:**
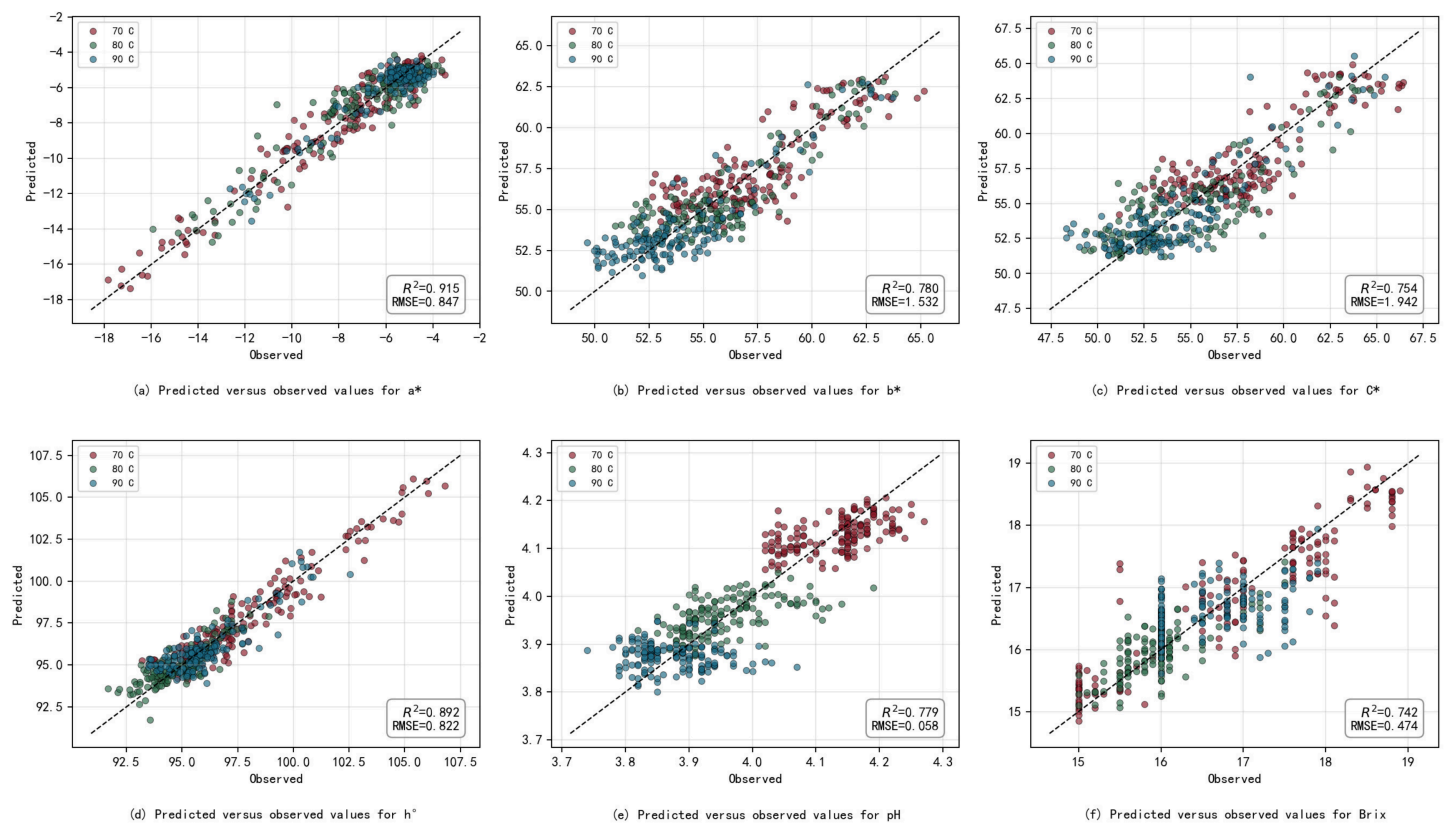
Final prediction graph of data fusion.

The observed-versus-predicted results of the PIRL–FiLM-ICM fusion model under LOSO validation, with samples distinguished by temperature, are shown in Figure 10. The model achieved R^2^ values of 0.915, 0.780, 0.754, 0.892, 0.779, and 0.742 for a*, b*, C*, h°, pH, and Brix, respectively. Predictions for a* and h° were closely distributed around the 1:1 line, whereas greater dispersion was observed for b*, C*, pH, and particularly Brix. Temperature-dependent clustering remained visible for pH and Brix, reflecting differences among thermal-treatment conditions. Nevertheless, samples from all three temperatures generally followed the same prediction trend, indicating that the model captured the principal temperature-dependent variations.

The results were obtained using CARS–SPA wavelength selection and GPR regression rather than an SPA–GPR benchmark. The effect of the temperature–time optimization strategy was evaluated through the C0–C4 ablation configurations under the same feature-selection and regression framework. C0 served as the reference model without temperature and time information, while C4 integrated PIRL and FiLM-ICM conditional modulation. Therefore, this scatter plot illustrates the target-specific predictive performance of the complete C4 fusion model; the contribution of PIRL and FiLM-ICM should be interpreted together with the C0–C4 ablation results rather than through comparison with a separately selected SPA model.

**Figure 11:**
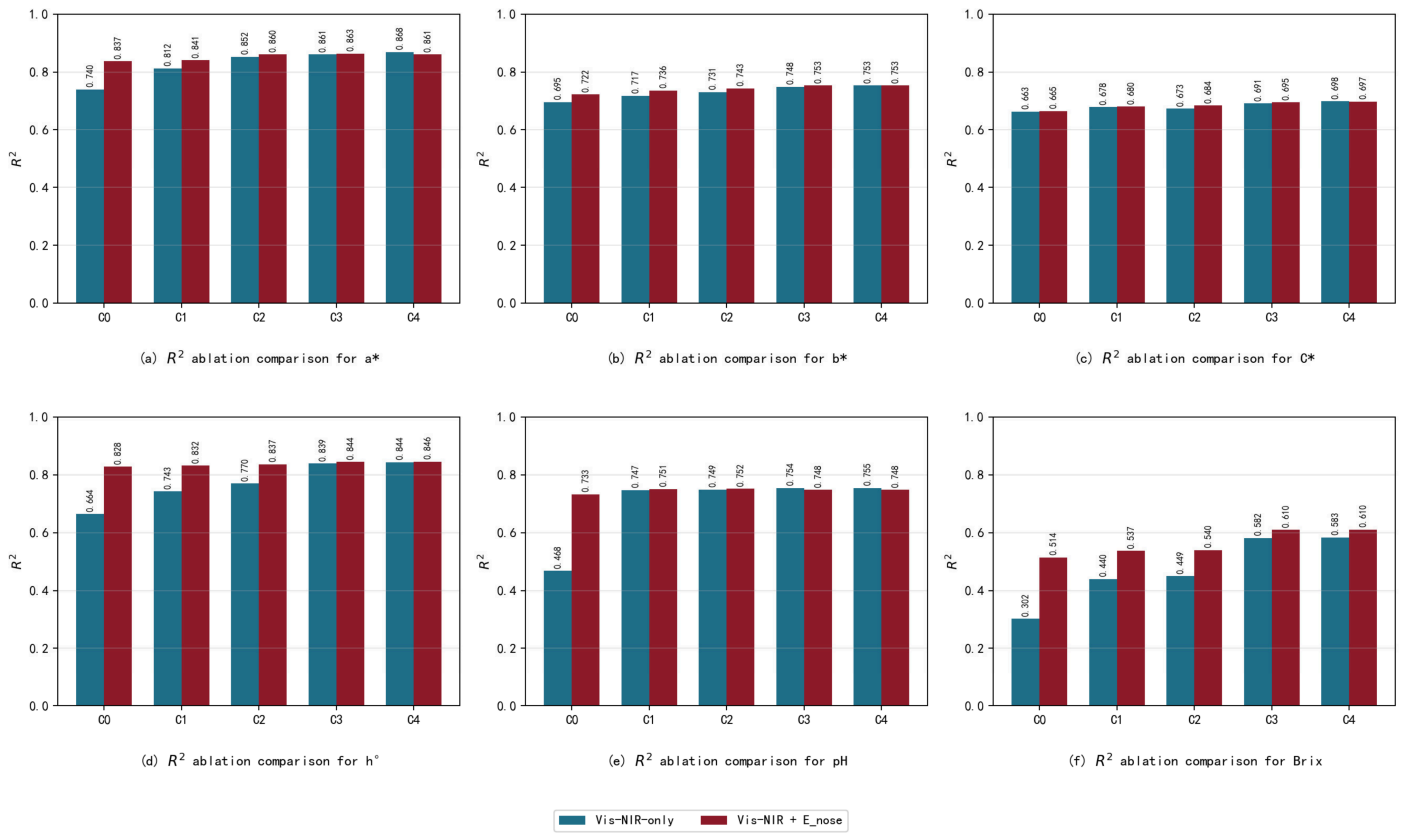
Comparison of R^2^ and RPD values for the CARS–SPA and GPR combinations.

Figure 11 compares the R^2^ and RPD values for the CARS–SPA and GPR combinations. The target-specific ablation results revealed that the benefits of the optimization strategies varied considerably among quality parameters. In the VIS–NIR-only path, the largest improvement from C0 to C4 was observed for Brix, whose R^2^ increased from 0.661 to 0.730. The R^2^ values of a* and h° also increased substantially, from 0.868 to 0.915 and from 0.840 to 0.886, respectively. In contrast, only limited improvements were obtained for C* (0.755 to 0.757), b* (0.780 to 0.783), and pH (0.765 to 0.781). These results demonstrate that the temperature–time optimization modules mainly benefited targets whose spectral relationships contained additional systematic variation that could be captured through physical-prior learning and conditional modulation.

After E-nose fusion, C0 already achieved relatively high R^2^ values for most targets, leaving less room for further improvement. From C0 to C4, the R^2^ values increased by 0.028 for Brix, 0.014 for a*, 0.008 for h°, and 0.003 for pH. However, no improvement was observed for C* and b*, whose R^2^ values decreased slightly by 0.001 and 0.010, respectively. Moreover, the differences between C3 and C4 were generally small, indicating that FiLM-ICM modulation captured most of the available temperature–time information and that adding PIRL produced only marginal target-dependent changes. Therefore, the optimization gain cannot be explained solely by the initial prediction accuracy; it depends more directly on the extent to which each target contains learnable temperature-dependent variation that is not already represented by the fused spectral and E-nose features.

### 3.6. Discussion

The first-order kinetic prior provides an interpretable trend term that captures the dominant direction of temperature-driven quality evolution. For color parameters (a*, b*, C*, h°), the kinetic assumption aligns reasonably well with the observed degradation patterns. This alignment arises because thermal degradation of pigments such as chlorophylls and carotenoids in kiwifruit juice approximately follows first-order behavior during early-to-mid-pasteurization stages. The kinetic prior supplies a physically meaningful baseline that constrains the machine learning model to deviate only when sample-specific spectral features indicate otherwise. As shown in Figure 6, the prior curves tracked the overall decline of color parameters, though they underestimated the rate at 90 °C, suggesting that higher temperatures may introduce additional degradation pathways not fully captured by a single-rate model.

For Brix and pH, however, the kinetic fit is inherently less accurate. Brix is influenced by at least two competing mechanisms: soluble solids concentration due to moisture evaporation, and dissolution or transformation of pectin and sugars. These processes are not strictly temperature-rate dependent in a simple first-order sense, and they may not leave strong signatures in the VIS–NIR spectral range. pH is even more challenging, as it reflects proton activity in a complex buffered system involving organic acids and their dissociation equilibria, which respond to thermal treatment nonlinearly. Paradoxically, this inaccuracy makes the residual-learning component more essential: the physical term captures the gross trend, while the machine learning model compensates for non-kinetic deviations. This explains why Brix and pH showed the largest RPD improvements (0.229 and 0.380, respectively) from C0 to C4, as reported in Section 3.5.

The FiLM-ICM-inspired conditional modulation enhances model performance by re-weighting spectral feature contributions according to the current thermal condition. Unlike simple concatenation of temperature and time as additional covariates (C1), FiLM-ICM actively reshapes the feature space, allowing the model to emphasize spectral bands that are more informative under specific temperature regimes (Figure 7). This mechanism proves particularly beneficial for parameters with strong temperature sensitivity, such as a* and h°, where the modulation effectively amplifies the spectral signatures associated with pigment degradation while suppressing temperature-independent noise. Conversely, for parameters like pH, whose variation arises from buffering systems rather than direct thermal degradation of chromophores, the spectral responses are intrinsically weaker, and hence FiLM-ICM modulation yields more limited gains. The target-specific ablation results (Section 3.5) confirm this interpretation: a* and h° improved substantially with FiLM-ICM (C3) compared with C1, whereas pH and C* showed only marginal changes. This differential effect is not a shortcoming but rather a diagnostic feature: it indicates that FiLM-ICM is not indiscriminately improving all predictions, but selectively enhancing those with a stronger spectral–thermal coupling.

A notable finding from the ablation study is that the combined PIRL–FiLM-ICM configuration (C4) did not exceed FiLM-ICM alone (C3) in terms of R^2^ for most targets. Two explanations are plausible. First, information overlap: once FiLM-ICM modulates sensor features using temperature–time conditioning, the physical trend provided by PIRL may encode largely the same temperature-dependent information, leading to redundancy rather than complementarity. Second, performance saturation: with CARS–SPA feature selection already retaining the most informative spectral variables, the baseline model (C0) already achieves a mean R^2^ of 0.803 after E-nose fusion, leaving limited room for further improvement. This finding itself is valuable: it suggests that when baseline sensor features are already sufficiently informative, additional physical or modulation modules yield diminishing returns, which is a practically important insight for model design in small-sample scenarios.

Comparison with deep learning approaches: While deep learning methods such as convolutional neural networks or Transformers have shown impressive performance in large-scale spectral analysis, their applicability to small-sample food processing studies is constrained by high data demand and computational overhead. In the present dataset of 456 samples, a conventional deep learning model would likely overfit unless heavily regularized or pretrained. By contrast, the proposed PIRL–FiLM-ICM framework requires no large-scale training data or GPU resources; the entire modeling and validation pipeline runs on standard computing hardware. More importantly, the physical prior parameters retain clear engineering interpretations, enabling process engineers to relate model outputs directly to degradation kinetics, which is rarely possible with black-box deep networks. Thus, for small-sample quality monitoring applications, the proposed framework offers a pragmatic balance between prediction accuracy, interpretability, and computational efficiency.

Limitations and future work: The results, while encouraging, should be interpreted within the scope of the experimental design. This study used a single kiwifruit cultivar from one harvest batch and laboratory-scale pasteurization conditions. The eight replicates were technical rather than biological replicates, meaning that the reported R^2^ values reflect model performance under controlled conditions and may not fully capture natural variability. Additionally, the E-nose system was custom-built for this study; different sensor configurations would require adjustments to feature extraction and model parameters. The method’s transferability to other food matrices would require retraining with new kinetic parameters. From an application perspective, real-time industrial deployment would necessitate optimization of the feature extraction pipeline and integration with edge-computing hardware, as the current offline analysis has not yet been validated in an online setting. Future work should include external validation on independent batches and different cultivars to assess generalization, as well as exploration of zero-order or Weibull kinetic models for targets where first-order kinetics provide inadequate fits. As noted in Section 2.2, the dataset of 456 samples from 24 trajectories is modest. Although CARS–SPA feature selection, LOSO cross-validation, and the PIRL prior were employed to mitigate overfitting, the small sample size limits statistical power and the assessment of batch-to-batch variability.

## 4. Conclusions

Overall, this study has demonstrated that in small-sample VIS–NIR time-series prediction tasks, model performance improvement does not rely solely on more complex architectures, but can be effectively achieved through the synergistic coupling of physical priors, conditional information, and spectral features. Specifically, embedding the volatilization–saturation kinetic equation as a physical prior has provided an interpretable trend term for temperature-driven color evolution while transforming the modeling paradigm from pure data fitting to a residual learning framework with mechanistic constraints. Complementarily, the introduction of FiLM-ICM conditional modulation has elevated temperature and time from low-dimensional covariates simply concatenated with high-dimensional spectral features to regulatory factors that actively participate in feature expression, thereby enhancing model adaptability across different thermal conditions and processing stages.

Compared with deep neural network-based approaches, the proposed framework is particularly well-suited for agricultural product quality detection scenarios characterized by limited sample sizes and high experimental costs. The physical prior parameters carry clear engineering interpretations, substantially improving the interpretability of model predictions. Quantitative evaluations under strict leave-one-trajectory-out cross-validation confirmed that the PIRL–FiLM-ICM configuration consistently outperformed baseline models across all quality indicators, with the most substantial generalization gains observed for Brix and pH (RPD increases of 0.229 and 0.380, respectively).

Nevertheless, the findings should be interpreted with caution, as they are based on a single kiwifruit cultivar under laboratory-controlled conditions with a relatively small dataset. Future work should include external validation on independent batches and different cultivars, exploration of transfer learning for cross-product adaptation, and validation under industrial-scale and real-time deployment scenarios.

## Figures and Tables

**Table 1:** Compact vertical comparison table.

Configuration	VIS–NIR-Only	VIS–NIR + E-Nose
**Temporal window**	t − 1, t − 2 → t	t − 1, t − 2 → t
**NIR features**	40 variables × 2 = 80	40 variables × 2 = 80
**E-nose features**	—	16 sensors (mean/SD/slope) → 6 PCs/point × 2 = 12
**Auxiliary variables**	Temperature, sampling time, interval	Temperature, sampling time, interval
**Input dimension**	83	95

**Table 2:** Performance comparison of different feature selection methods across five regression models (R^2^).

Feature Method	GPR	XGB	RF	SVR	PLS
CARS–SPA	0.7934	0.7707	0.7637	0.7753	0.7149
UVE	0.7816	0.7682	0.7627	0.7743	0.7034
CARS	0.7537	0.7527	0.7435	0.7463	0.6828
SPA	0.7665	0.7602	0.7513	0.7485	0.6490
VIP	0.7182	0.7134	0.7057	0.6965	0.5946
Corr	0.6895	0.6886	0.6813	0.6671	0.5775
iPLS	0.6803	0.7037	0.7152	0.5843	0.4951

**Table 3:** Performance comparison of different feature selection methods across five regression models (RPD).

Feature Method	GPR	XGB	RF	SVR	PLS
CARS–SPA	2.29	2.16	2.10	2.21	1.99
UVE	2.23	2.15	2.12	2.20	1.94
CARS	2.09	2.11	2.02	2.05	1.88
SPA	2.16	2.12	2.07	2.08	1.76
VIP	1.91	1.90	1.87	1.84	1.65
Corr	1.87	1.87	1.82	1.79	1.62
iPLS	1.83	1.96	1.97	1.64	1.47

## Data Availability

The original contributions presented in this study are included in the article. Further inquiries can be directed to the corresponding author.
